# Development and validation of bioimpedance prediction equations for fat-free mass in unilateral male amputees

**DOI:** 10.7717/peerj.10970

**Published:** 2021-03-08

**Authors:** Hyuk-Jae Choi, Chang-Yong Ko, Yunhee Chang, Gyoo-Suk Kim, Kyungsik Choi, Chul-Hyun Kim

**Affiliations:** 1 Department of Medical Convergence Research & Development, Rehabilitation Engineering Research Institute, Incheon, Republic of Korea; 2 Department of Research & Development, Refind Inc, Wonju, Gangwon-do, Republic of Korea; 3 Department of Healthcare Business Division, Healthmax company, Seoul, Gangnam-gu, Republic of Korea; 4 Department of Sports Medicine, Soonchunhyang University, Asan, Chungcheongnam-do, Republic of Korea

**Keywords:** Bioelectrical impedance analysis, Dual-energy X-ray absorptiometry, Clinical diagnosis, Amputation, Body composition, Multiple regression analysis, Estimated regression equation, Fat-free mass

## Abstract

**Background:**

Metabolic disease due to increased fat mass is observed in amputees (APTs), thereby restricting their activity. Systemic health management with periodic body composition (BC) testing is essential for healthy living. Bioelectrical impedance analysis (BIA) is a non-invasive and low-cost method to test BC; however, the APTs are classified as being exempted in the BIA.

**Objective:**

To develop segmental estimated regression equations (sEREs) for determining the fat-free mass (FFM, kg) suitable for APTs and improve the accuracy and validity of the sERE.

**Methods:**

Seventy-five male APTs participated in this cross-sectional study. Multiple regression analysis was performed to develop highly accurate sEREs of BIA based on independent variables derived from anthropometric measurements, dual-energy X-ray absorptiometry (DXA), and BIA parameters. The difference in validity between the predicted DXA and sum of the segmentally-predicted FFM values by sEREs (Sum_sEREs) values was evaluated using bivariate linear regression analysis and the Bland–Altman plot.

**Results:**

The coefficient of determination (*R^2^*) and total error (*TE*) between DXA and Sum_sEREs were 71% and 5.4 (kg) in the cross-validation analysis.

**Conclusions:**

We confirmed the possibility of evaluating the FFM of APTs through the sEREs developed in this study. We also identified several independent variables that should be considered while developing such sEREs. Further studies are required to determine the validity of our sEREs and the most appropriate BIA frequencies for measuring FFM in APTs.

## Introduction

Amputation refers to the removal of the upper or lower extremities to resolve the cause of a disability due to disease or trauma (Hecht, MedicineNet.com & Shiel, 2003). Physical and functional constraints are more severe in amputees (APTs) than in the general population (Coffey, Gallagher & Desmond, 2014). Previously, most amputations were performed following war-related or occupational injuries. Indeed, an increase in the number of APTs was observed following World War II and the Iraq War (Kulkarni et al., 1998; Robbins et al., 2009).

In addition, cardiovascular diseases such as high blood pressure/stroke and metabolic diseases including diabetes mellitus may necessitate amputation (Kopelman, 2000; Rahman & Berenson, 2010). Currently, reports suggest that the rate of amputation continues to increase worldwide. Notably, the number of APTs in the United States is predicted to increase from approximately 1.6 million (2008 data) to 3.6 million by 2050 (Ziegler-Graham et al., 2008).

Diabetes is associated with impaired blood supply to the extremities, which may lead to necrosis or severe ulcers that necessitate amputation (Mishra et al., 2017). Post-amputation complications, such as atrophy, phantom limb pain, and contracture, may lead to further activity constraints beyond those imposed by the amputation, thereby exerting deleterious effects on one’s overall health (Gallagher, Allen & Maclachlan, 2001; O’Sullivan et al., 2019; Ustun et al., 2003). Additional post-amputation complications include obesity or excessive fat accumulation (Kurdibaylo, 1996), atrophy due to decreased muscle mobilization for ambulation and joint stability, and decreased physical strength (Centomo et al., 2008; Isakov et al., 1996; Renstrom, Grimby & Larsson, 1983a; Renstrom et al., 1983b; Sadeghi, Allard & Duhaime, 2001; Bukowski, 2006; Zachariah et al., 2004).

These complications may lead to an overall deterioration in physical health, which can be reflected by changes in BC and muscle condition, ultimately resulting in a decreased quality of life (Chin et al., 2002; Rosenberg et al., 2013; Suk, Bom & Do, 2001). Periodic assessments of BC and weight management interventions are critical for preventing secondary complications of amputation, such as excessive body fat/obesity (Stone et al., 2006; Yoo, 2014). Such assessments and interventions may not only help prevent obesity and muscle atrophy, but may also improve overall physical/mental health and quality of life.

Dual-energy X-ray absorptiometry (DXA) allows the measurement of bone mineral content (BMC), fat mass (FM), and soft lean tissue mass (SLTM) by passing two X-ray beams through the body. Although DXA is a highly accurate, validated method for the assessment of fat-free mass (FFM) (i.e., BMC+SLTM), these assessments are time-consuming and expensive. Furthermore, DXA requires exposure to small amounts of radiation, and some participants may be uncomfortable given the limited measurement space. Despite these limitations, DXA continues to be widely touted as the gold standard for determining BC (Janssen, Heymsfield & Ross, 2002; Lee & Gallagher, 2008; Woodrow, 2009).

Commercially available bioimpedance devices include single- and multi-frequency impedance analysis (SFBIA and MFBIA) and bioimpedance spectroscopy (BIS) (Mulasi et al., 2015). The principle of bioelectrical impedance analysis (BIA), including SFBIA, MFBIA, and BIS methods is to determine electrical impedance as “resistance (*R*)” (Kyle et al., 2004a) for total body water (TBW) and “reactance (*Xc*)” for body cell mass (Walter-Kroker et al., 2011). BIA is advantageous in that it is faster, more useful, less invasive, less physically restrictive, and less expensive than DXA. In addition, BIA equipment occupies far less space and requires less effort to operate than DXA equipment with a non-portable nature, which limits its use (Buckinx et al., 2015; Janssen, Heymsfield & Ross, 2002). As a routine clinical BIA tool, BC analysis was now readily available in a wider range of clinics and in the community (Ward, 2019). Given these advantages, BIA is performed in various settings. To estimate BC, BIA devices measure TBW content by sending a micro-current of less than 800 µA throughout the body. An estimated regression equation (ERE) is then used to predict FM and FFM (kg) (Kyle et al., 2004a). Recently, BIA has been used for the quantitation of BC through a specific mathematical model without empirically derived variables for athletic players (Sardinha et al., 2020) (Campa & Toselli, 2018; Toselli et al., 2020), children (Colica et al., 2018), older adults (Silveira et al., 2020), and young patients with cystic fibrosis (Charatsi et al., 2016).

However, APTs are typically excluded from such BIA studies owing to differences in limb length between the amputated and sound sides and irregular limb shapes (Dogan et al., 2012). Since the heights of the two sides differ, it is against the impedance index (*ZI*) formula (height^2^/impedance), which is a more significant single predictor of FFM than other anthropometric variables (Nguyen et al., 2007).

In general, BC is predicted using the wrist-ankle method and variables, which consider the whole-body height for reducing typical BIA errors. Thus, the use of BIA equations, which are based on individuals (Beaudart et al., 2020) without amputation may not be accurate and valid when measuring the BC of APTs.

Therefore, we intended to develop EREs of APT using the variable of ZI and considering the segmental length based on Tanaka’s study (Tanaka et al., 2007). Appropriate segmental estimated regression equation (sERE) for BC in APTs should consider residual limb properties, such as length, various other factors as independent variables including age, height, weight, gender, *ZI*, *R*, *Xc*, and phage angle (*PA*) without segmental amputated limb weight that is not measurable.

In this study, the InBody S10 BIA instrument (InBody S10) was used. This instrument allows for segmental analysis of various cellular properties through frequencies. Adhere-type electrodes are placed at eight precise tactile points to perform BIA in a comfortable and safe supine position for ATPs.

In this study, DXA measurements were used as a reference standard to develop EREs for BIA of APTs. The development of a valid ERE for use in APTs may improve their health management by providing accurate and convenient assessments of FFM through BIA. We confirmed the possibility of evaluating FFM of APTs through the sERE developed in this study. For an overview of abbreviations and parameters, see Table 1.

**Table 1:** Abbreviations and concepts.

Abbreviation	Description
General parameters:	
BC	Body composition
BIA	Bioimpedance analysis
BIS	Bioimpedance spectroscopy
BPL	Body part length
DXA	Dual-energy X-ray absorptiometry
ERE	Estimated regression equations
MFBIA	Multi-frequency bioimpedance analysis
ROI	Regions of interest
sEREs	Segmental estimated regression equations
SFBIA	Single-frequency bioimpedance analysis
Sum_sEREs	Sum of the segmentally-predicted FFM values by sEREs
Subjects parameters:	
APTs	Amputees
LA, RA, LL, RL, TR	Left arm (LA), Right arm (RA), Left leg (LL), Right leg (RL), Trunk (TR)
Physiological parameters:	
BMC	Bone mineral content (kg)
FFM	Fat-free mass (kg)
FM	Fat mass (kg)
SLTM	Soft lean tissue mass (kg)
TBW	Total body water (L)
BIA parameters:	
*PA*	Phage angle (^∘^)
*R*	Resistance (Ohm, Ω)
*Xc*	Reactance (Ohm, Ω)
*Z*	Impedance (Ohm, Ω).
*ZI*	Impedance index (ZI=Height^2^/Z)
Statistical analysis parameters:	
LOA	Limits of agreement
*R*^2^	Coefficient of determination
SEE	Standard error of estimate
*TE*	Total error
VIF	Variance inflation factor

## Materials & Methods

### Participants

This study was approved by the Institutional Review Board (No. 1040875-201707-SB-030 and RERI-IRB-190924-1). After receiving a complete description of the study, all participants provided written informed consent. A total of 75 male, unilateral APTs were recruited and included in the study. The mean age of the participants was 43.6 ± 12 years. Seventeen participants had previously undergone upper limb amputation (trans-humeral amputation: *n* = 5; trans-radial amputation: *n* = 12), and 58 had previously undergone lower limb amputation (trans-femoral amputation: *n* = 32; trans-tibial amputation: *n* = 26). APTs with disarticulation and multilateral (bi-, tri-) amputation were excluded. Additional participant characteristics including residual limb length (cm) and onset (postoperative period, years) are presented in Table 2.

**Table 2:** Participant characteristics.

	Upper-limb APTs (*n* = 17)		Lower-limb APTs (*n* = 58)	
	Trans-humeral (*n* = 5)	Trans-radial (*n* = 12)	Trans-femoral (*n* = 32)	Trans-tibial (*n* = 26)
Age (year)	42.6 ± 5.7^*^	50.4 ± 11.6	41.1 ± 13.3	43.6 ± 10.5
Height (cm)	174.6 ± 4.2	168.3 ± 7.2	172.0 ± 5.9	171.4 ± 5.6
Weight (kg)	78.7 ± 4.0	74.2 ± 7.6	73.7 ± 13.7	73.2 ± 12.5
BMI (kg/m)	25.9 ± 2.3	26.2 ± 2.0	24.8 ± 3.8	24.9 ± 3.6
Residual limb Length (cm)	21.4 ± 3.1	42.7 ± 4.9	34.6 ± 6.2	62.2 ± 6.7
Onset (year)	13.6 ± 7.9	16.8 ± 11.8	13.2 ± 11.9	18.2 ± 13.9

**Notes.**

* Mean ± SD; APTs, amputees; BMI, body mass index; SD, standard deviation.

### Experimental device (DXA)

DXA (Lunar Corp., Madison, WI, USA) measurements of FFM were used as the reference standard in the development of the ERE. This instrument was calibrated through the spine phantom provided by the manufacture daily. To standardize the scan, files from the original DXA system were transferred to iDXA software, version 4.0.2. The scan process was blinded and fulfilled by one radiologist who wore protective clothing. The segmentation method based on Heymsfield et al. (1990) was applied to uniform measurement (Heymsfield et al., 1990).

Participants were instructed to wear comfortable clothing for the assessment. Under the guidance of a professional examiner, each participant was asked to lay comfortably in the supine position on the assessment table, and spread both the upper and lower limbs. The whole-body DXA was performed for approximately 15 min.

### Experimental device (BIA)

**Figure 1:**
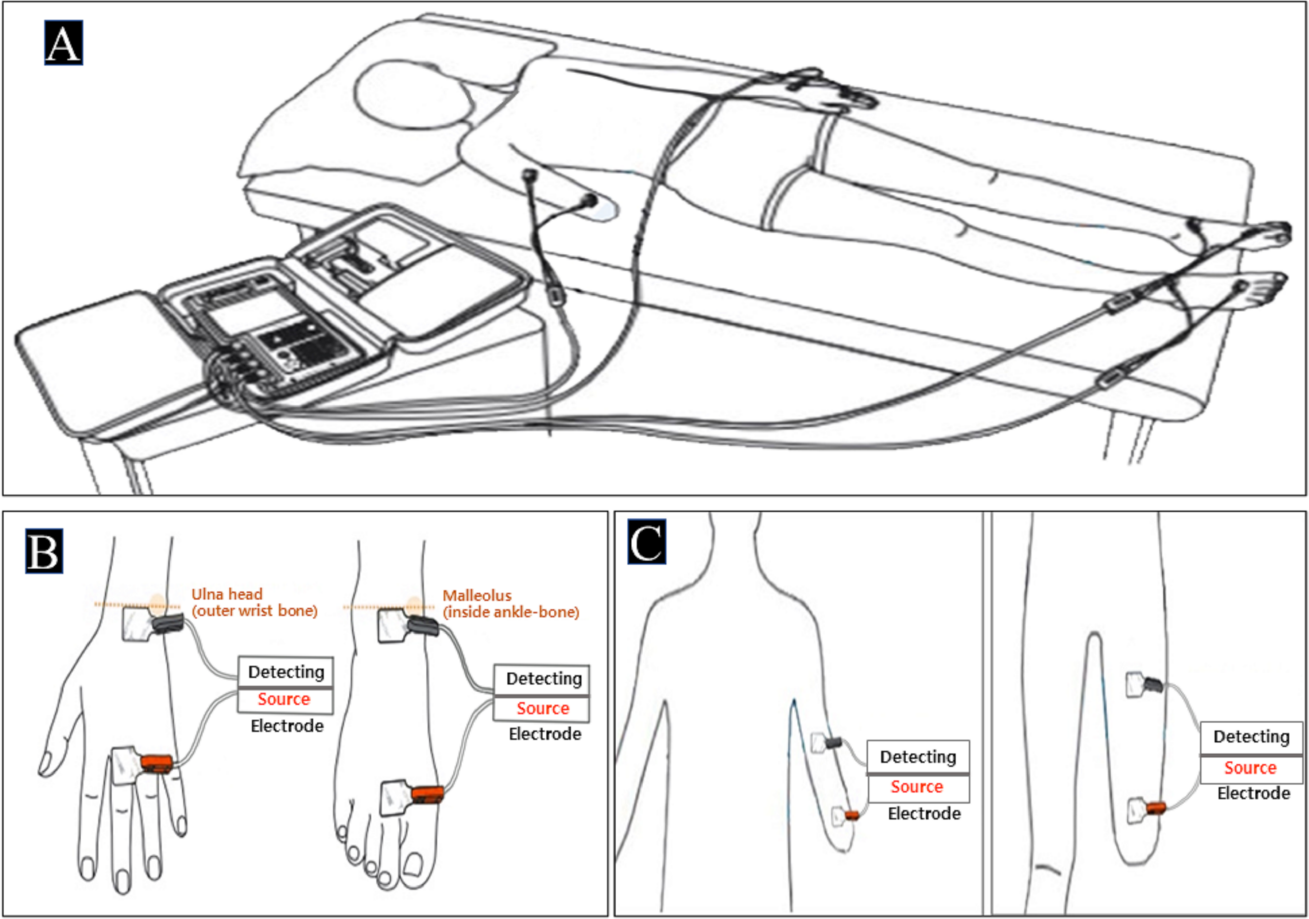
The testing postures and the electrode placements. (A) InBody S10 in the supine position [permitted from the manufacture] (B) Electrode placements of sound limbs (C) Electrode placements of residual limbs.

BIA of the tetrapolar 8-point electrode type (InBody S10 for the supine measure, InBody Co. South Korea) was used in this study. This BIA model uses eight electrodes positioned at each hand and foot and enables multifrequency impedance measurement of the arms, trunk, and legs. Impedance parameters were measured with alternating current of 80 and 100 mA at frequencies of 1, 5, 50, 250, 500, and 1,000 kHz for InBody S10. After checking for precision errors of FFM about repeatability through biplicate measurements of the same APT based on a previous study (Buckinx et al., 2015), the InBody S10 was used. It was designed for single measurements in the supine position on a non-conductive surface through a SFBIA of only 50 kHz. In the sound limb, defined anatomical sites were cleaned with alcohol, on which adhesive gel electrodes were placed on the dorsal surfaces of the hand, wrist, ankle, and foot as follows: the proximal edge of the wrist electrode was attached from an imaginary line bisecting the styloid process of the ulna and the proximal edge of the finger electrode on an imaginary line from the imaginary line bisecting the metatarsophalangeal joint of the middle finger. The proximal edge of the ankle electrode was attached from an imaginary line bisecting the medial malleolus and the distal edge of the toe electrode was placed from an imaginary line through the metatarsophalangeal joints of the second toe as shown in Fig. 1B. In the residual limb, the distal and proximal electrodes were attached from the end of the stump (=distal part) to the region (=proximal part) by keeping the distance according to the instructions of InBody S10. Additionally, fixed-distance of electrodes was used with a 5-cm standard distance as shown in Fig. 1C (Kaysen et al., 2005; Kriemler et al., 2009). The device was calibrated every morning using the standard control circuit supplied by the manufacturer. We confirmed that the precision error was less than 2% (Fig. 1).

### Definition of segmental ZI (ZI) and regions of interest (ROI) in DXA

Impedance indices (*ZI*) for each body part were determined considering the residual limb length in each participant. *ZI* values were calculated in accordance with methods described by Tanaka et al. (2007). As shown in Eq. (1), *ZI* was calculated by dividing height^2^ by *Z* (based on values for non-APTs). We then calculated the body part length *ZI* (*ZI*_BPL_) by dividing the body part length (BPL)^2^ by Z. (Tanaka et al., 2007). BPL was measured for the following five areas: left arm (LA), right arm (RA), left leg (LL), right leg (RL), and trunk (TR). The reference positions for the lengths of the upper/lower limbs and trunk were defined as follows: the area between the humeral head (=acromion) and styloid processes of the wrist (ulna) for the upper limbs; the area from the anterior superior iliac spine to the medial malleoli for the lower limbs; and the posterior length between the seventh cervical vertebra prominens to the center of the posterior superior iliac spine for the trunk (Beattie et al., 1990; Hackenberg et al., 2003; Jamaluddin et al., 2011; Lee, Cha & Lee, 2016; Neelly, Wallmann & Backus, 2013; Ross, 1972). The results of the experiment were analyzed by matching the physical measurements to the BIA electrode locations and DXA ROI based on these measurement standards.

According to the Heymsfield’s protocol (1990), the boundaries of the ROI are defined as follows: (1) for the upper limbs of the ROI (right and left), the arms are isolated by running a line through the humeral head and (2) for the lower limbs, the pelvis cut is placed just above the pelvic brim and the computer automatically draws the lower pelvic lines to bisect the hip joints (Heymsfield et al., 1990; Jeon et al., 2020).

(1) \begin{eqnarray*}ZI= \frac{{H}^{2}}{Z} \end{eqnarray*}

H: Height of the whole body, Z: Impedance

(2) \begin{eqnarray*}Z{I}_{BPL}= \frac{BP{L}^{2}}{Z} \end{eqnarray*}

BPL: Body part length, Z: Impedance

### Independent variables for segmental BIA

Independent variables included in the EREs were determined for the five body areas as follows: *R* was applied by differentiating among the LA (*R*_LA_), RA (*R*_RA_), LL (*R*_LL_), RL (*R*_RL_), and trunk (*R*_TR_). Using notations identical to those for *R*, impedance (*Z*), reactance (*Xc*), and PA were also calculated for each body part and expressed in terms of *R*_BPL_, *Z*_BPL_, *Xc*_BPL_, and *PA*_BPL_.

### Experimental procedures

Prior to the measurements of FFM, participants were instructed to abstain from excessive dehydration-accompanied exercises and excessive alcohol use. In addition, they were instructed to fast for at least 6 h and abstain from alcohol consumption for at least 4 h.

We ensured that the urinary bladder was voided in all participants within 30 min before measurement, all participants wore non-conductive and comfortable sportswear. All conductive materials, prosthetic limbs, and amputation covers (silicone, amputation protection, etc.) were removed. First, DXA and then BIA test was conducted. For the measurements, after lying on a DXA sheet, stability (stabilization of BC) measurement was taken for 5 min in the supine position considering the potential for variable fluid shifts, and the DXA scan was then performed, and BIA was measured on the place immediately after the DXA scan without changing the position. The shoulder and hip joints of all participants were maintained at an abduction angle of approximately 15° for the shoulder and hip joints. The elbow and knee joints were extended in a straight anatomical position. The physical contact of each electrode was ensured in accordance with the criteria recommended by the manufacturer. Each measurement took approximately 5–15 min. Participants were instructed to maintain a comfortable position without any movement during the examination (Brantlov et al., 2017; Kyle et al., 2004d).

### Sum of the segmentally-predicted FFM values by sERE (Sum_sEREs)

After the development of the sEREs of the five body parts in the APTs, the sum of the segmentally-predicted FFM values by sERE was calculated. We confirmed the validity and accuracy between DXA and the sum of sERE_BIA about FFM through bivariate linear regression and the Bland_Altman plot.

### Statistical analysis

The physical characteristics of the APTs group are presented as means with SDs. A normality test is required if there are fewer than 30 participants. However, since the number of participants in our study was 75, we assumed normality and analyzed all data (Kwak & Kim, 2017; Sang Gyu et al., 2019). The forward stepwise multiple linear regression analysis was used to develop sEREs in the APTs group. The significance level was set to *p* < 0.05. Variables included in the initial analyses contained *ZI*_*BPL*_, *R*_*BPL*_, *Xc*_*BPL*_, *PA*_*BPL*_, age (yr), height (cm), and weight (kg). The developmental equations were selected by measures of goodness-of-fit statistics, including coefficient of determination (*R*^2^), the standard error of estimate (SEE), acceptable subjective rating of SEE (i.e., good to excellent) according to the minimally acceptable standard for prediction errors (Buckinx et al., 2018; Heyward & Wagner, 2004a; Heyward & Wagner, 2004b; Lohman, 1992), and the variance inflation factor (VIF). The SEE measures the variation in the actual values from the predicted values. The SEE represents the degree of deviation of individual scores form the regression line. It is computed using the following formula:

SEE = $\sqrt{\sum (Measured \mathrm{FFM}-Estimated \mathrm{FFM})^{2}/(N-p-1)}$

where *p* = number of predicter variables. The VIF assesses how much the variance of an estimated regression coefficient increases when predictors are correlated for estimating collinearity/multicollinearity. In case of values more than 10, it can be assumed that the regression coefficients are poorly estimated due to multi-collinearity to remove predictors from the model. In our study, with values less than 10 (Lee et al., 2018; Wickramasinghe et al., 2008), we could proceed with our regression analysis. In the cross-validation, the group predictive accuracy of the Sum_sEREs was tested by calculating *R*^2^, total error (*TE*: The TE represents the degree of deviation from the line of identity using the formula:

Total Error = $\sqrt{\sum (Measured \mathrm{FFM}-Estimated \mathrm{FFM})^{2}/N}$),

and acceptable subjective rating of TE (Heyward & Wagner, 2004a; Heyward & Wagner, 2004b; Lohman, 1992). The individual predictive accuracy of these equations was also tested by Bland-Altman plots, whitch included the bias of the mean difference between measured values of DXA and predicted values of Sum_sEREs. We used the 95% limits of agreement (LOA) between equations, and concordance correlation efficient (*r*_*y*−*y*′,*mean*_). Data were analyzed using Microsoft Office Excel Ver. 2013 (Microsoft, Redmond, WA, USA) and SPSS version 18.0 (IBM, USA).

## Results

### Segmental Estimated Regression Equation

DXA measurements of FFM were used as the dependent variable in the development of the EREs for use in APTs. Various independent variables were entered to ensure optimal model development. Our model considered factors such as *R*^2^, multicollinearity (tolerance and variance inflation factor [VIF]), and standard error estimates (SEE). Using these factors, we developed sEREs for the left and right upper/lower limbs as well as the trunk. *ZI*_LA_, *Xc*_LA_, height, and age were entered as independent variables in the final sERE model for FFM in the left arm (LA_FFM_). Values for the final sERE for LA_FFM_ were as follows: *R* = 0.95, *R*^2^ = 0.90, and adjusted *R*^2^ = 0.89. *ZI*_RA_ and *Xc*_RA_ were entered as independent variables in the final sERE model for FFM in the right arm.

Values for the final sERE for RA _FFM_ were as follows: *R* = 0.86, *R*^2^ = 0.74, and adjusted *R*^2^ = 0.73. *R*_BPL_, *Xc*_BPL_, *ZI*_BPL_, and weight were entered as independent variables in the final models for both the left and right lower limbs. The LL_FFM_ model included *R*_LL_, *Xc*_LL_, *ZI*_LL_, and weight. The final sERE values for LL_FFM_ were as follows: *R* = 0.95, *R*^2^ = 0.91, and adjusted *R*^2^ = 0.90. The highest correlation coefficients were observed for RL_FFM_: *R* = 0.97, *R*^2^ = 0.94, and adjusted *R*^2^ = 0.93. In contrast, the lowest correlation coefficients were observed for TR_FFM_: *R* = 0.88, *R*^2^ = 0.78, and adjusted *R*^2^ = 0.76. A total of five independent variables were entered for the TR_FFM_ ERE: *ZI*_TR_, weight, height, age, and *R*_TR_ (Table 3).

**Table 3:** The Final segmental estimated regression equations for FFM (kg).

LA_FFM_	*y* = − 3.759 + 0.204(ZI_LA_) + 0.410(Xc_LA_) + 0.019(height) - 0.007(age)			
	*R* = .948	*R*^2^ = .898	Adj. *R*^2^ = .892	SEE = 0.286
	VIF: ZI_LA_ = 1.286, Xc_LA_ = 1.193, height = 1.378, age = 1.274			
RA_FFM_	*y* = − 1.370 + 0.212(ZI_RA_) + 0.054(Xc_RA_)			
	*R* = .858	*R*^2^ = .736	Adj. *R*^2^ = .729	*SEE* = 0.402
	VIF: ZI_RA_ = 1.002, Xc_RA_ = 1.002			
LL_FFM_	*y* = − 4.089 + 0.162(Xc_LL_) + 0.143(ZI_LL_) + 0.039(weight) + 0.006(R_LL_)			
	*R* = .953	R^2^ = .908	Adj. R^2^ = .902	SEE = 0.909
	VIF: Xc_LL_ = 4.712, ZI_LL_ = 1.593, weight = 1.451, R_LL_ = 4.465			
RL_FFM_	*y* = − 3.715 + 0.009 (R_RL_) + 0.152(ZI_RL_) + 0.139(Xc_RL_) + 0.031(weight)			
	*R* = .968	*R*^2^ = .937	Adj. *R*^2^ = .933	SEE = 0.739
	VIF: R_RL_ = 8.942, ZI_RL_ = 1.460, Xc_RL_ = 8.415, weight = 1.166			
TR_FFM_	*y* = − 12.061 + 0.046(ZI_TR_) + 0.073(weight) + 0.212(height) –0.419(R_TR_) + 0.041(age)			
	*R* = .880	*R*^2^ = .775	Adj. R^2^ = .758	SEE = 1.510
	VIF: ZI_TR_ = 2.441, weight = 1.649, height = 1.823, R_TR_ = 1.709, age = 1.294			

**Notes.**

FFM fat-free mass (kg) ZI_BPL_ body part length impedance index Z_BPL_ impedance Xc_BPL_ reactance R_BPL_ resistance LA left arm RA right arm TR trunk LL left leg RL right leg Adj. Adjusted VIF variation inflation factor SEE standard error estimate (kg)

### Cross-validation between BIA and DXA about FFM

#### Linear regression, total error, and line of identity

Linear regression analyses were used to calculate the correlation between BIA estimates based on the final segmental ERE and standard DXA measurements. In the total error (*TE*) calculation (Eq. (3)), Y–Y’ represents the difference between the DXA measurement (Y) and the BIA estimate (Y’), while N represents the sample size.

(3) \begin{eqnarray*}TE=\sqrt{\sum { \left( \mathrm{Y }-\mathrm{Y ` } \right) }^{2}/N}\end{eqnarray*}

The *TE* for the comparison between DXA and BIA values for FFM was 5.4 (kg), with a correlation of 0.84 (*p* <  0.05) (Fig. 1).

### Residuals and bias in Bland–Altman plots

**Figure 2:**
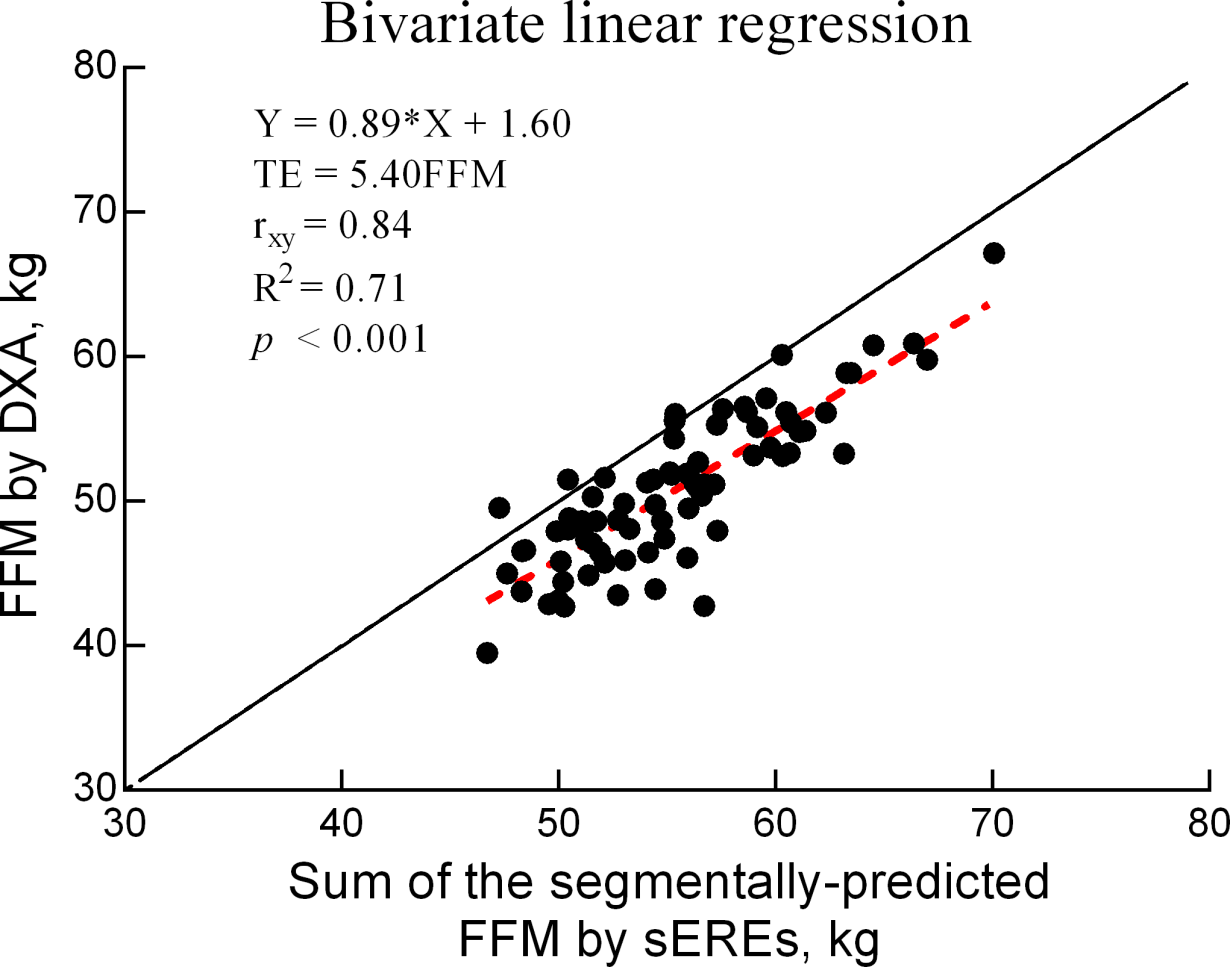
Bivariate linear regression for FFM values obtained using DXA and sEREs. FFM: fat-free mass (kg), sEREs: segmental estimated regression equations, TE: total error, r: validity coefficient, DXA: dual-energy X-ray absorptiometry.

To evaluate the validity of the final ERE, the residuals (i.e., the difference between BIA-estimated and DXA-measured FFM) and means of the two methods were assessed using the Bland–Altman plot (Fig. 2). The bias of the difference between the two FFM measurements was –4.60 kg. BIA estimates obtained using the ERE tended to be higher than those obtained using DXA values. Furthermore, there was a tendency for the residuals of the bias to be evenly distributed, and increased bias tended to be associated with decreased residuals.

## Discussion

In the present study, we aimed to develop sERE for BIA in APTs using DXA measurements as the reference standard. Given the correlation (*r* = 0.85), *TE* (5.4 kg), and coefficient of determination (*R*^2^ = 71%) between FFM values obtained using Sum_sEREs and DXA, our findings confirmed that the sERE developed in this study could assess FFM in APTs.

Based on findings obtained in previous studies, sex differences should be considered in the selection of independent variables to obtain more accurate estimates (Beaudart et al., 2020). However, this study was conducted on only male APTs because of a difference in the sex ratio within the total APTs recruited; hence, representativeness of sex could not be achieved. Furthermore, data from the Multidimensional Body–Self Relations Questionnaire suggest that women experience significantly higher level of dissatisfaction with their bodies than do men following amputation (Holzer et al., 2014). For the above reasons, our sEREs were developed using data from male APTs only.

The process of developing sERE was carried out by applying the FFM of DXA on the same limb BIA variables. Therefore, the dominant and non-dominant limbs were not considered specifically. Additionally, in the process of validation of the developed sERE for each limb, the same limb variables were applied. For example, the DXA value of the right leg of the amputated side limb and the BIA of the same right limb were compared.

There are examples for various types of amputations for uni-, bi-, and multi-lateral APTs. However, in this study, for the development of a basic sERE, only unilateral APTs were included to control the length variable of the amputated site. Moreover, given the difficulty in controlling for the BPL among multilateral APTs, we restricted our participants to unilateral APTs.

The measurement range of the InBody S10 device (Biospace, Korea) extends from 5 to 1,000 kHz (1, 5, 50, 250, 500, and 1,000 kHz). MFBIA can more accurately measure intra- and extracellular body water than SFBIA of 50 kHz. However, SFBIA of 50 kHz is better for measuring cell membrane properties through Xc, because it provides equivalence of information for the function of Xc at 50 kHz versus other frequencies (Piccoli et al., 2005). For developing basic sERE of APTs, we carefully analyzed the cell membrane state of the APT rather than analyzing between the intra- and extracellular body water properties (Kyle et al., 2004b; Heymsfield et al., 2005). Only an SFBIA of 50 kHz was used for the calculation of total body water, on which estimations for FFM are based using proprietary equations (Achamrah et al., 2018).

Previous BIA studies have excluded APTs as well as patients with joint deformation, hemi-paralysis, and uncommonly large/small bodies (Dogan et al., 2012; Kyle et al., 2004e; Mialich, Sicchieri & Junior, 2014). Tanaka et al. (2007) proposed using a tetrapolar 4-point BIA to measure BC in such individuals (Mialich, Sicchieri & Junior, 2014; Tanaka et al., 2007).

However, in a 4-point BIA with the tetrapolar system, the electrodes are attached manually by connecting electrodes to measure the segments according to manufacturer’s instructions. Four-point estimates are derived based on the characteristics of the flow of current on one side of the body, allowing for the calculation of BC for the specific body part, following which total estimates are obtained. Thus, the measurements had substantial systematic errors, including underestimation or overestimation of accuracy (Janssen et al., 2000; Tanaka et al., 2007). Given the errors of the tetrapolar 4-point BIA, Foster and Lukaski (1996) highlighted the need for further research regarding the use of such measurements for the analyses of BC (Lukaski, 1996). To avoid these issues, we utilized the tetrapolar 8-point segmental BIA (sBIA) as InBody S10, in which the pairs of electrodes are attached to measure the different body segments.

Before 1980, the ERE for FFM only included the resistance index (height^2^/resistance). *ZI* was calculated by taking into account the variables for the whole-body height in ERE of non-APTs. However, the sERE of APT developed in this study was analyzed by applying *ZI*_*BPL*_ and BIA parameters considering the length of each body part, including the residual limbs and condition of this amputated region.

In five sEREs for each part, the redundancy of variables was considered through VIF and the highly accurate sEREs calculated through a meticulous analysis process based on the BIA characteristics that affect the APT.

The BIA variables applied to the sEREs are *R, Xc*, and *Z* as shown in Table 3. we were to confirm that a complex quantity composed of (*R*) which is caused by TBW, and capacitance of the cell membrane related to (*Xc*), and obstruction to the flow of an alternating current (*Z*) that was dependent on the frequency of the applied current based on the theoretical basis of BIA in the limbs of APT (Kyle et al., 2004a; Kyle et al., 2004c) (Khalil, Mohktar & Ibrahim, 2014).

Generally, assuming our body as a cylinder, both arms and legs are attached to the body, and the whole-body height is used for the *ZI* (*ZI* =Height^2^/Z). However, in this APT study, the length of each limb was substituted for the *ZI* of each limb (*ZI*_BPL_=BPL^2^/*Z*) to make the estimation equation of the segmental limb considering amputated extremity. For example, in the process of developing estimating equations, such as the FFM of each of the right upper and left upper limbs etc., the final model with a low statistical error and a high estimating power was selected using the length and BIA variables of the same body part. The APTs had imbalance between the left and right limbs, similar to that reported in a previous study (Sherk, Bemben & Bemben, 2010), showing muscle and fat imbalance between the injured and sound limb; consequently, there was a difference in variables entered in the final sERE obtained from the final model selected as the criterion of the significance level (*P*<0.05), VIF (<10), SEE, and *R*^2^.

In the present study, we utilized a forward stepwise multiple regression analysis that included diverse independent variables such as residual limb length. When developing an ERE, approximately 20 participants are required for each independent variable entered. Given that our study included 75 participants, four or fewer independent variables are considered appropriate (Heyward & Wagner, 2004a; Heyward & Wagner, 2004b). These independent variables included ZI_BPL_, height, weight, age, onset, and segmental BIA factors.

Table 3 shows the final EREs. The number of variables entered in each sERE ranged from two to five. Although previous studies have specified that only four variables should be included, based on our sample size, it was necessary to consider factors, such as *R*^2^, multicollinearity (tolerance and VIF), and SEE, to develop the most ideal model. Nonetheless, the sERE for the trunk was the only equation to have been developed using five variables.

BIA estimates of FFM are based on TBW measurements, which are derived from *Z* values obtained by passing microcurrents throughout the human body. In this calculation, the human body is assumed to be cylindrical (TBW = *ρ* x height^2^/Z, *ρ*=constant). Based on the theory that TBW comprises 73% of FFM (TBW = FFM × 0.73), the estimates of FFM can be obtained using the following equation: FFM = TBW/0.73. Factors, such as race, age, sex, and medical history, influence the unique conduction constant (*ρ*) as well as the correlation between TBW and *ZI*, making it necessary to include several independent variables in the TBW calculation (Kyle et al., 2004b). Therefore, in this study, we included additional independent variables, such as onset and characteristics of the body part amputated.

Several BIA studies conducted outside of Korea have included patients with hemi-paralysis (Kafri, Potter & Myint, 2014; Nalepa et al., 2019; Yoo et al., 2016), pediatric scoliosis (Matusik, Durmala & Matusik, 2016), or Turner’s syndrome (i.e., abnormally small body)(Guedes et al., 2010). However, no such studies have been conducted on the APTs. Despite this, studies have recommended that BIA measurements be obtained in the non-amputated limb (Kyle et al., 2004e; Mialich, Sicchieri & Junior, 2014).

However, to estimate the whole-body FFM, the amputated body parts must be considered. In our study, the sEREs for FFM did not exhibit a close relationship with *PA*_BPL_, and we did not include PA as an independent variable for any ERE in Table 3, similar to models developed using data from the general population (Mialich, Sicchieri & Junior, 2014). Meanwhile, *Xc* has been utilized in numerous EREs for FFM in studies conducted outside Korea (Lieberman, 1993; Kyle et al., 2001; Roubenoff et al., 1997; Stolarczyk et al., 1994).

In accordance with previous findings, our sERE for FFM exhibited a close relationship with *Xc*, whereas onset did not appear to exert a significant impact on FFM estimates. Although changes in BC occur over time following amputation, FFM can be maintained with systematic rehabilitation and BC management during the first postoperative year. This was noted by a previous study of those for whom 2 to 15 years had passed since amputation (Eckard et al., 2015; Renstrom et al., 1983b).

In our study, the final sEREs were selected by considering factors, such as *R*^2^, tolerance, VIF, and SEE. As shown in Fig. 2, the correlation for FFM values was high (*R* = 0.84), with an *R*^2^ of 71%. This correlation is higher than the standard of 0.80 (*R*^2^ = 64%) suggested by Heyward and Wagner (Heyward & Wagner, 2004a; Heyward & Wagner, 2004b) for validity research. A bivariate linear regression equation (Y = aX ±  b) was used to confirm the accuracy of Sum_sEREs as well as the correlation. The slope (a) and y-intercept (b) were used to analyze the correlation between DXA and BIA measurements. The slope of the bivariate linear regression equation was 0.89, whereas the y-intercept was 1.60, yielding a simply positive correlation of y = x. However, the slope of 0.89 exhibited a positive correlation that was close to the standard of 1. Furthermore, the y-intercept was close to the ideal standard of 0. Taken together, these results suggest the possibility of sERE for APT.

**Figure 3:**
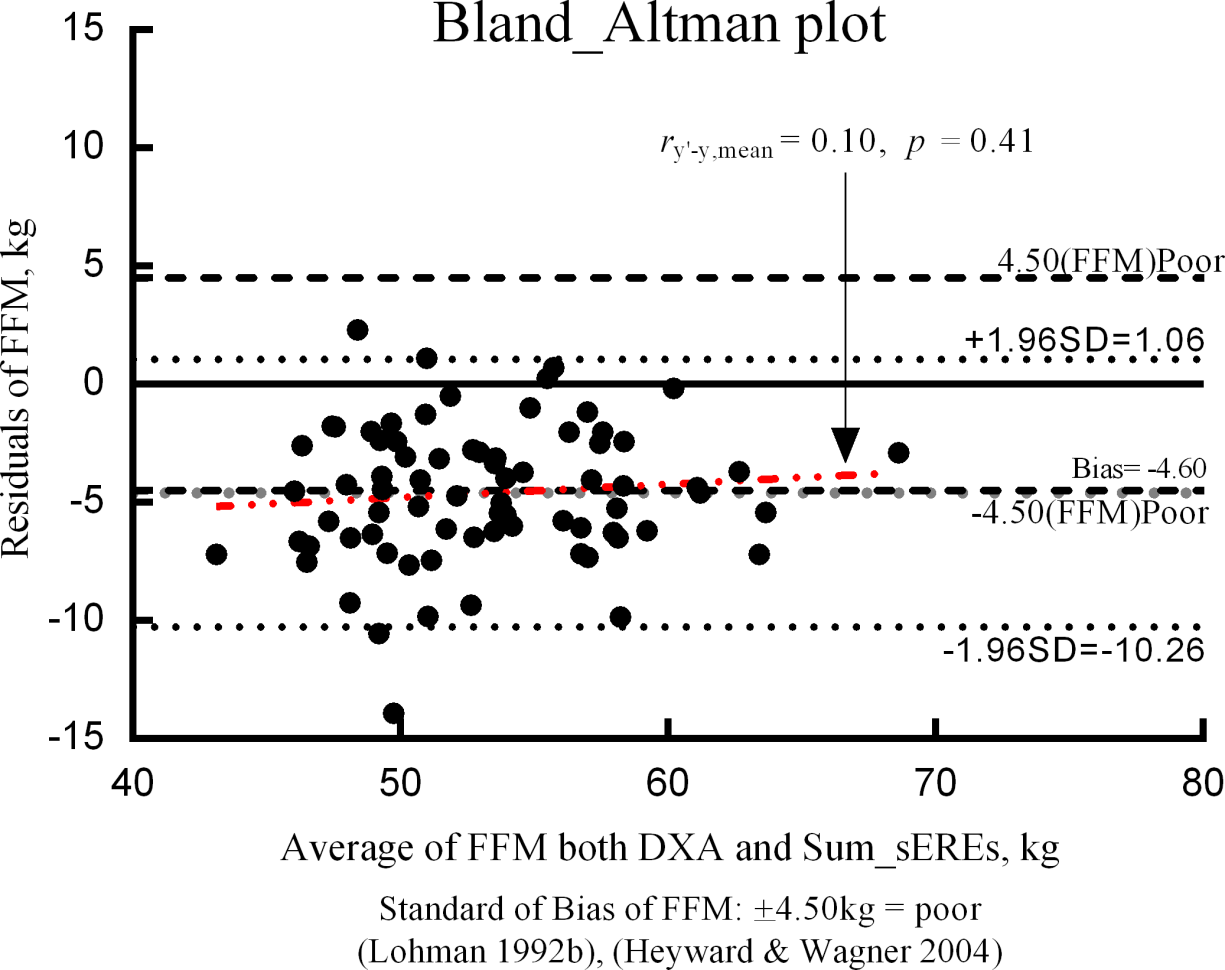
Bland–Altman plot. Bias: mean of DXA-BIA value, ± 4.5(FFM) poor = “poor” standard for evaluating prediction errors, FFM: fat-free mass (kg), DXA: dual-energy X-ray absorptiometry, sEREs: segmental estimated regression equations.

Individual errors for DXA and BIA results are shown in the Bland–Altman plot in Fig. 3. The average value representing the difference between the two methods (i.e., Bias) was -4.60 kg. When the bias approaches 0 ( *y* = 0), there is no mean difference in the measurement values with an ideal validity. However, bias did not approach 0 in our study. BIA tended to overestimate FFM, relative to the value obtained using DXA. Ainsworth et al. (1997) suggested that the results are ideal if the proportion that exceeds the standard of overestimation and underestimation is less than 30% (Ainsworth et al., 1997). In our study, the proportion of APTs who exceeded the standard of bias was 50.7%, suggesting a need for further validity studies.

### Limitation

In this study, limitations were placed on the use of only 50kHz frequency to characterize the cell membrane; however, ICF and ECF have limitations in reflecting the characteristics at a normal level. We intend to proceed with the MFBIA study including 1, 50, 250, 500, 1000kHz, and over frequencies, as a future research project. In the validation procedure, we recognized the importance of validating a method through a split group design, K-fold or LOOV-type. However, the sEREs were developed without external cross-validation through group of predictive power test. We confirmed only cross-validation between DXA and Sum_sEREs of FFM values. Additionally, we also did not perform a thoughtful analysis of the PA, but we intend to do it for the APTs in the future.

## Conclusions

In the present study, we utilized multiple regression analysis to develop sEREs for FFM in APTs, using DXA as the reference standard. Although there was a bias of −4.598, and LOA of −0.26 1.06 in our results, we could confirm the minimal clinical feasibility based on the coefficient of determination (*R*^2^ = 71%), and *TE* (5.40 kg). In addition, we identified several independent variables that should be considered while developing such sEREs for APT. Further studies are required to determine the validity of our sEREs and the most appropriate BIA frequencies for measuring FFM in APTs.

##  Supplemental Information

10.7717/peerj.10970/supp-1 Supplemental Information 1 Raw data

## Additional Information and Declarations

### Data Availability

The following information was supplied regarding data availability: Raw data are available as a Supplementary File.

### Clinical Trial Registration

The following information was supplied regarding Clinical Trial registration: No. 1040875-201707-SB-030 and RERI-IRB-190924-1.

## References

[ref-1] Achamrah N, Colange G, Delay J, Rimbert A, Folope V, Petit A, Grigioni S, Déchelotte P, Coëffier M (2018). Comparison of body composition assessment by DXA and BIA according to the body mass index: a retrospective study on 3655 measures. PLOS ONE.

[ref-2] Ainsworth BE, Stolarczyk LM, Heyward VH, Berry CB, Irwin ML, Mussulman LM (1997). Predictive accuracy of bioimpedance in estimating fat-free mass of African-American women. Medicine and Science in Sports and Exercise.

[ref-3] Beattie P, Isaacson K, Riddle DL, Rothstein JM (1990). Validity of derived measurements of leg-length differences obtained by use of a tape measure. Physical Therapy.

[ref-4] Beaudart C, Bruyère O, Geerinck A, Hajaoui M, Scafoglieri A, Perkisas S, Bautmans I, Gielen E, Reginster JY, Buckinx F (2020). Equation models developed with bioelectric impedance analysis tools to assess muscle mass: a systematic review. Clinical Nutrition ESPEN.

[ref-5] Brantlov S, Ward LC, Jødal L, Rittig S, Lange A (2017). Critical factors and their impact on bioelectrical impedance analysis in children: a review. Journal of Medical Engineering and Technology.

[ref-6] Buckinx F, Landi F, Cesari M, Fielding RA, Visser M, Engelke K, Maggi S, Dennison E, Al-Daghri NM, Allepaerts S, Bauer J, Bautmans I, Brandi ML, Bruyère O, Cederholm T, Cerreta F, Cherubini A, Cooper C, Cruz-Jentoft A, McCloskey E, Dawson-Hughes B, Kaufman JM, Laslop A, Petermans J, Reginster JY, Rizzoli R, Robinson S, Rolland Y, Rueda R, Vellas B, Kanis JA (2018). Pitfalls in the measurement of muscle mass: a need for a reference standard. Journal of Cachexia, Sarcopenia and Muscle.

[ref-7] Buckinx F, Reginster JY, Dardenne N, Croisiser JL, Kaux JF, Beaudart C, Slomian J, Bruyère O (2015). Concordance between muscle mass assessed by bioelectrical impedance analysis and by dual energy X-ray absorptiometry: a cross-sectional study. BMC Musculoskeletal Disorders.

[ref-8] Bukowski EL (2006). Atlas of amputations and limb deficiencies: surgical, prosthetic, and rehabilitation principles, ed 3. Physical Therapy.

[ref-9] Campa F, Toselli S (2018). Bioimpedance vector analysis of elite, subelite, and low-level male volleyball players. International Journal of Sports Physiology and Performance.

[ref-10] Centomo H, Amarantini D, Martin L, Prince F (2008). Differences in the coordination of agonist and antagonist muscle groups in below-knee amputee and able-bodied children during dynamic exercise. Journal of Electromyography & Kinesiology.

[ref-11] Charatsi AM, Dusser P, Freund R, Maruani G, Rossin H, Boulier A, Le Bourgeois M, Chedevergne F, De Blic J, Letourneur A, Casimir G, Jais JP, Sermet-Gaudelus I (2016). Bioelectrical impedance in young patients with cystic fibrosis: validation of a specific equation and clinical relevance. Journal of Cystic Fibrosis.

[ref-12] Chin T, Sawamura S, Fujita H, Nakajima S, Oyabu H, Nagakura Y, Ojima I, Otsuka H, Nakagawa A (2002). Physical fitness of lower limb amputees. American Journal of Physical Medicine & Rehabilitation.

[ref-13] Coffey L, Gallagher P, Desmond D (2014). Goal pursuit and goal adjustment as predictors of disability and quality of life among individuals with a lower limb amputation: a prospective study. Archives of Physical Medicine and Rehabilitation.

[ref-14] Colica C, Di Renzo L, Gualtieri P, Romano L, Costade Miranda R, De Lorenzo A, Purificato I (2018). Development and cross-validation of predictive equation for estimating total body lean in children. Annali Dell Istituto Superiore di Sanita.

[ref-15] Dogan MH, Karadag B, Ozyigit T, Kayaoglu S, Ozturk AO, Altuntas Y (2012). Correlations between sarcopenia and hypertensive target organ damage in a Turkish cohort. Acta Clinica Belgica.

[ref-16] Eckard CS, Pruziner AL, Sanchez AD, Andrews AM (2015). Metabolic and body composition changes in first year following traumatic amputation. Journal of Rehabilitation Research and Development.

[ref-17] Gallagher P, Allen D, Maclachlan M (2001). Phantom limb pain and residual limb pain following lower limb amputation: a descriptive analysis. Disability and Rehabilitation.

[ref-18] Guedes AD, Bianco B, Lipay MV, Callou EQ, Castro ML, Verreschi IT (2010). A specific bioelectrical impedance equation to predict body composition in Turner’s syndrome. Arq Bras Endocrinol Metabol.

[ref-19] Hackenberg L, Hierholzer E, Potzl W, Gotze C, Liljenqvist U (2003). Rasterstereographic back shape analysis in idiopathic scoliosis after posterior correction and fusion. Clinical Biomechanics (Bristol, Avon).

[ref-20] Hecht F, Shiel WC, MedicineNet.com (2003). Webster’s new world medical dictionary.

[ref-21] Heymsfield S, Lohman T, Wang ZM, Going SB (2005). Human body composition.

[ref-22] Heymsfield SB, Smith R, Aulet M, Bensen B, Lichtman S, Wang J, Pierson Jr RN (1990). Appendicular skeletal muscle mass: measurement by dual-photon absorptiometry. American Journal of Clinical Nutrition.

[ref-23] Heyward VH, Wagner DR (2004a). Applied body composition assessment.

[ref-24] Heyward VH, Wagner DR (2004b). Applied body composition assessment.

[ref-25] Holzer LA, Sevelda F, Fraberger G, Bluder O, Kickinger W, Holzer G (2014). Body image and self-esteem in lower-limb amputees. PLOS ONE.

[ref-26] Isakov E, Burger H, Gregoric M, Marincek C (1996). Stump length as related to atrophy and strength of the thigh muscles in trans-tibial amputees. Prosthetics and Orthotics International.

[ref-27] Jamaluddin S, Sulaiman AR, Imran MK, Juhara H, Ezane MA, Nordin S (2011). Reliability and accuracy of the tape measurement method with a nearest reading of 5 mm in the assessment of leg length discrepancy. Singapore Medical Journal.

[ref-28] Janssen I, Heymsfield SB, Baumgartner RN, Ross R (2000). Estimation of skeletal muscle mass by bioelectrical impedance analysis. Journal of Applied Physiology.

[ref-29] Janssen I, Heymsfield SB, Ross R (2002). Application of simple anthropometry in the assessment of health risk: implications for the Canadian physical activity, fitness and lifestyle appraisal. Journal of Applied Physiology.

[ref-30] Jeon KC, Kim SY, Jiang FL, Chung S, Ambegaonkar JP, Park JH, Kim YJ, Kim CH (2020). Prediction equations of the multifrequency standing and supine bioimpedance for appendicular skeletal muscle mass in Korean older people. International Journal of Environmental Research and Public Health.

[ref-31] Kafri MW, Potter JF, Myint PK (2014). Multi-frequency bioelectrical impedance analysis for assessing fat mass and fat-free mass in stroke or transient ischaemic attack patients. European Journal of Clinical Nutrition.

[ref-32] Kaysen GA, Zhu F, Sarkar S, Heymsfield SB, Wong J, Kaitwatcharachai C, Kuhlmann MK, Levin NW (2005). Estimation of total-body and limb muscle mass in hemodialysis patients by using multifrequency bioimpedance spectroscopy. American Journal of Clinical Nutrition.

[ref-33] Khalil SF, Mohktar MS, Ibrahim F (2014). The theory and fundamentals of bioimpedance analysis in clinical status monitoring and diagnosis of diseases. Sensors.

[ref-34] Kopelman PG (2000). Obesity as a medical problem. Nature.

[ref-35] Kriemler S, Puder J, Zahner L, Roth R, Braun-Fahrländer C, Bedogni G (2009). Cross-validation of bioelectrical impedance analysis for the assessment of body composition in a representative sample of 6- to 13-year-old children. European Journal of Clinical Nutrition.

[ref-36] Kulkarni J, Adams J, Thomas E, Silman A (1998). Association between amputation, arthritis and osteopenia in British male war veterans with major lower limb amputations. Clinical Rehabilitation.

[ref-37] Kurdibaylo SF (1996). Obesity and metabolic disorders in adults with lower limb amputation. Journal of Rehabilitation Research and Development.

[ref-38] Kwak SG, Kim JH (2017). Central limit theorem: the cornerstone of modern statistics. Korean Journal of Anesthesiology.

[ref-39] Kyle UG, Bosaeus I, De Lorenzo AD, Deurenberg P, Elia M, Gomez JM, Heitmann BLs, Kent-Smith L, Melchior JC, Pirlich M, Scharfetter H, Schols AM, Pichard C (2004d). Bioelectrical impedance analysis-part II: utilization in clinical practice. Clinical Nutrition.

[ref-40] Kyle UG, Bosaeus I, De Lorenzo AD, Deurenberg P, Elia M, Gomez JM, Heitmann BL, Kent-Smith L, Melchior JC, Pirlich M, Scharfetter H, Schols AM, Pichard C, Espen P (2004e). Bioelectrical impedance analysis-part II: utilization in clinical practice. Clinical Nutrition.

[ref-41] Kyle UG, Bosaeus I, De Lorenzo AD, Deurenberg P, Elia M, Gómez JM, Heitmann BL, Kent-Smith L, Melchior JC, Pirlich M, Scharfetter H, Schols AMWJ, Pichard C (2004b). Bioelectrical impedance analysis - Part I: review of principles and methods. Clinical Nutrition.

[ref-42] Kyle UG, Bosaeus I, De Lorenzo AD, Deurenberg P, Elia M, Gómez JM, Heitmann BL, Kent-Smith L, Melchior JC, Pirlich M, Scharfetter H, Schols AMWJ, Pichard C (2004c). Bioelectrical impedance analysis - Part II: utilization in clinical practice. Clinical Nutrition.

[ref-43] Kyle UG, Bosaeus I, De Lorenzo AD, Deurenberg P, Elia M, Gomez JM, Heitmann BL, Kent-Smith L, Melchior JC, Pirlich M, Scharfetter H, Schols AM, Pichard C, Compositionof the EWG (2004a). Bioelectrical impedance analysis–part I: review of principles and methods. Clinical Nutrition.

[ref-44] Kyle UG, Genton L, Karsegard L, Slosman DO, Pichard C (2001). Single prediction equation for bioelectrical impedance analysis in adults aged 20–94 years. Nutrition.

[ref-45] Lee SY, Ahn S, Kim YJ, Ji MJ, Kim KM, Choi SH, Jang HC, Lim S (2018). Comparison between dual-energy x-ray absorptiometry and bioelectrical impedance analyses for accuracy in measuring whole body muscle mass and appendicular skeletal muscle mass. Nutrients.

[ref-46] Lee BJ, Cha HG, Lee WH (2016). The effects of sitting with the right leg crossed on the trunk length and pelvic torsion of healthy individuals. Journal of Physical Therapy Science.

[ref-47] Lee SY, Gallagher D (2008). Assessment methods in human body composition. Current Opinion in Clinical Nutrition & Metabolic Care.

[ref-48] Lieberman LS (1993). Advances in body composition assessment, By Timothy G. Lohman. vii + 150 pp. Champaign, IL: Human Kinetics Publishers, 1992. $18.00 (paper). American Journal of Human Biology.

[ref-49] Lohman TG (1992). Advances in body composition assessment.

[ref-50] Lukaski HC (1996). Biological indexes considered in the derivation of the bioelectrical impedance analysis. American Journal of Clinical Nutrition.

[ref-51] Matusik E, Durmala J, Matusik P (2016). Association of body composition with curve severity in children and adolescents with idiopathic scoliosis (IS). Nutrients.

[ref-52] Mialich MS, Sicchieri JMF, Junior AAJ (2014). Analysis of body composition: a critical review of the use of bioelectrical impedance analysis. International Journal of Clinical Nutrition.

[ref-53] Mishra SC, Chhatbar KC, Kashikar A, Mehndiratta A (2017). Diabetic foot. Bmj.

[ref-54] Mulasi U, Kuchnia AJ, Cole AJ, Earthman CP (2015). Bioimpedance at the bedside: current applications, limitations, and opportunities. Nutrition in Clinical Practice.

[ref-55] Nalepa D, Czarkowska M, Zaluska W, Jakubowska K, Chrusciel P (2019). Electrical bioimpedance in patients after ischemic stroke, a civilization disease. Annals of Agricultural and Environmental Medicine.

[ref-56] Neelly K, Wallmann HW, Backus CJ (2013). Validity of measuring leg length with a tape measure compared to a computed tomography scan. Physiotherapy: Theory and Practice.

[ref-57] Nguyen QD, Fusch G, Armbrust S, Jochum F, Fusch C (2007). Impedance index or standard anthropometric measurements, which is the better variable for predicting fat-free mass in sick children?. *Acta Paediatrica*.

[ref-58] O’Sullivan SB, Schmitz TJ, Fulk G, O’Sullivan SB (2019). Physical Rehabilitation.

[ref-59] Piccoli A, Pastori G, Guizzo M, Rebeschini M, Naso A, Cascone C (2005). Equivalence of information from single versus multiple frequency bioimpedance vector analysis in hemodialysis. Kidney International.

[ref-60] Rahman M, Berenson AB (2010). Accuracy of current body mass index obesity classification for white, black, and Hispanic reproductive-age women. Obstetrics and Gynecology.

[ref-61] Renstrom P, Grimby G, Larsson E (1983a). Thigh muscle strength in below-knee amputees. Scandinavian Journal of Rehabilitation Medicine.

[ref-62] Renstrom P, Grimby G, Morelli B, Palmertz B (1983b). Thigh muscle atrophy in below-knee amputees. Scandinavian Journal of Rehabilitation Medicine.

[ref-63] Robbins CB, Vreeman DJ, Sothmann MS, Wilson SL, Oldridge NB (2009). A review of the long-term health outcomes associated with war-related amputation. Military Medicine.

[ref-64] Rosenberg DE, Turner AP, Littman AJ, Williams RM, Norvell DC, Hakimi KM, Czerniecki JM (2013). Body mass index patterns following dysvascular lower extremity amputation. Disability and Rehabilitation.

[ref-65] Ross CA (1972). Guidelines for measurement of amputation stump length. Bulletin of Prosthetics Research:.

[ref-66] Roubenoff R, Baumgartner RN, Harris TB, Dallal GE, Hannan MT, Economos CD, Stauber PM, Wilson PW, Kiel DP (1997). Application of bioelectrical impedance analysis to elderly populations. Journals of Gerontology. Series A, Biological Sciences and Medical Sciences.

[ref-67] Sadeghi H, Allard P, Duhaime PM (2001). Muscle power compensatory mechanisms in below-knee amputee gait. American Journal of Physical Medicine & Rehabilitation.

[ref-68] Sang Gyu K, lt, sup, gt, lt, sup, gt, Sung-Hoon P, lt, sup, gt, lt, sup, gt (2019). Normality test in clinical research. Journal of Rheumatic Diseases.

[ref-69] Sardinha LB, Correia IR, Magalhães JP, Júdice PB, Silva AM, Hetherington-Rauth M (2020). Development and validation of BIA prediction equations of upper and lower limb lean soft tissue in athletes. European Journal of Clinical Nutrition.

[ref-70] Sherk VD, Bemben MG, Bemben DA (2010). Interlimb muscle and fat comparisons in persons with lower-limb amputation. Archives of Physical Medicine and Rehabilitation.

[ref-71] Silveira EA, Barbosa LS, Rodrigues APS, Noll M, De Oliveira C (2020). Body fat percentage assessment by skinfold equation, bioimpedance and densitometry in older adults. Archives of Public Health.

[ref-72] Stolarczyk LM, Heyward VH, Hicks VL, Baumgartner RN (1994). Predictive accuracy of bioelectrical impedance in estimating body composition of Native American women. American Journal of Clinical Nutrition.

[ref-73] Stone PA, Flaherty SK, Aburahma AF, Hass SM, Jackson JM, Hayes JD, Hofeldt MJ, Hager CS, Elmore MS (2006). Factors affecting perioperative mortality and wound-related complications following major lower extremity amputations. Annals of Vasular Surgery.

[ref-74] Suk S, Bom PS, Do KS (2001). Assessment of quality of life in lower limb amputees using short-Form 36. Annals of Rehabilitation Medicine.

[ref-75] Tanaka NI, Miyatani M, Masuo Y, Fukunaga T, Kanehisa H (2007). Applicability of a segmental bioelectrical impedance analysis for predicting the whole body skeletal muscle volume. Journal of Applied Physiology.

[ref-76] Toselli S, Marini E, Latessa PMaietta, Benedetti L, Campa F (2020). Maturity related differences in body composition assessed by classic and specific bioimpedance vector analysis among male elite youth soccer players. International Journal of Environmental Research and Public Health.

[ref-77] Ustun TB, Chatterji S, Bickenbach J, Kostanjsek N, Schneider M (2003). The international classification of functioning, disability and health: a new tool for understanding disability and health. Disability and Rehabilitation.

[ref-78] Walter-Kroker A, Kroker A, Mattiucci-Guehlke M, Glaab T (2011). A practical guide to bioelectrical impedance analysis using the example of chronic obstructive pulmonary disease. Nutrition Journal.

[ref-79] Ward LC (2019). Bioelectrical impedance analysis for body composition assessment: reflections on accuracy, clinical utility, and standardisation. European Journal of Clinical Nutrition.

[ref-80] Wickramasinghe VP, Lamabadusuriya SP, Cleghorn GJ, Davies PSW (2008). Assessment of body composition in Sri Lankan children: validation of a bioelectrical impedance prediction equation. European Journal of Clinical Nutrition.

[ref-81] Woodrow G (2009). Body composition analysis techniques in the aged adult: indications and limitations. Current Opinion in Clinical Nutrition & Metabolic Care.

[ref-82] Yoo C, Kim J, Yang Y, Lee J, Jeon G (2016). Bioelectrical impedance analysis for severe stroke patients with upper extremity hemiplegia. Journal of Physical Therapy Science.

[ref-83] Yoo S (2014). Complications following an amputation. Physical Medicine and Rehabilitation Clinics of North America.

[ref-84] Zachariah SG, Saxena R, Fergason JR, Sanders JE (2004). Shape and volume change in the transtibial residuum over the short term: preliminary investigation of six subjects. Journal of Rehabilitation Research and Development.

[ref-85] Ziegler-Graham K, MacKenzie EJ, Ephraim PL, Travison TG, Brookmeyer R (2008). Estimating the prevalence of limb loss in the United States: 2005 to 2050. Archives of Physical Medicine and Rehabilitation.

